# eHealth Interventions to Treat Substance Use in Pregnancy: A Systematic Review and Meta-Analysis

**DOI:** 10.3390/ijerph18199952

**Published:** 2021-09-22

**Authors:** Katherine Silang, Hangsel Sanguino, Pooja R. Sohal, Charlie Rioux, Hyoun S. Kim, Lianne M. Tomfohr-Madsen

**Affiliations:** 1 Department of Psychology, University of Calgary, Calgary, AB T2N 1N4, Canada; katherine.silang@ucalgary.ca (K.S.); hangsel.sanguino@ucalgary.ca (H.S.); pooja.sohal@ucalgary.ca (P.R.S.); 2 Department of Educational Psychology and Leadership, Texas Tech University, Lubbock, TX 79409, USA; charlie.rioux@ttu.edu; 3 Department of Psychology, Ryerson University, Toronto, ON M5B 2K3, Canada; andrewhs.kim@ryerson.ca; 4 Alberta Children’s Hospital Research Institute (ACHRI), Calgary, AB T3B 6A8, Canada; 5 Department of Pediatrics, University of Calgary, Calgary, AB T2N 1N4, Canada

**Keywords:** pregnancy, substance-related disorders, randomized controlled trials, smoking, alcohol, cannabis, drug use, internet intervention, telemedicine, digital intervention

## Abstract

Substance use during pregnancy is associated with adverse pregnancy and neonatal outcomes; eHealth interventions offer a potential accessible treatment option. The objective of this systematic review and meta-analysis was to evaluate the effectiveness of eHealth interventions for the treatment of substance use during pregnancy. A comprehensive search of PsycINFO, Medline, CINAHL, Cochrane and Embase databases was conducted from May 2020 to April 2021. The protocol for this study was registered with Prospero (CRD42020205186) through the University of York Centre for Reviews and Dissemination. Two independent reviewers completed screening, data extraction, and quality assessment. RCTs were included if they reported: (a) administration of an eHealth intervention for (b) substance use outcomes, among (c) pregnant individuals. Comprehensive Meta-Analysis Software (CMA) was used to calculate pooled effect sizes (Odds Ratio) to determine the effect of eHealth interventions on substance use outcomes. Six studies were identified with substance use outcomes that included: smoking (*n* = 3), alcohol (*n* = 2), and other (*n* = 1). eHealth interventions were delivered through the internet (*n* = 1), computer (*n* = 3), telephone (*n* = 1), and text (*n* = 1). Results suggested that eHealth interventions significantly reduced substance use in pregnant individuals compared to controls (OR = 1.33, *95%* CI = 1.06 to 1.65, *p* = 0.013). eHealth interventions offer a promising and accessible treatment option to reduce substance use during pregnancy. This work was supported by the generous donors of the Alberta Children’s Hospital Foundation, the Canadian Child Health Clinician Scientist Program (CCHCSP), the Canadian Institute of Health Research and the Fonds de Recherche du Québec—Santé.

## 1. Introduction

Heavy substance use is associated with serious physical and psychological consequences [1]. The risk of developing a substance use disorder is heightened during reproductive years [2] and substance use is prevalent during pregnancy. 11–15% of pregnant individuals reporting use of alcohol, tobacco, cannabis and/or illicit substances [3,4,5]. The actual prevalence may be higher, as stigma and judgement may cause some pregnant people to be hesitant to report substance use [6]. 

Heavy substance use in pregnancy has serious short and long-term consequences, including elevated risk of miscarriage [7], low birthweight [8], infant mortality [9], and sudden infant death syndrome [10]. Long term outcomes for children exposed to substances in-utero vary [11]. For example, prenatal cannabis use has been linked to reduced attention and executive functioning skills, poor academic achievement, and increased behavioural problems [12]. Prenatal drinking has been linked to multiple long-term effects including cognitive and behavioural issues [13], executive functioning deficits [14], and poor psychosocial outcomes [15].

Given the high prevalence of substance use in pregnancy and its serious associated harms, it is imperative that pregnant individuals receive access to evidence-based supports. Despite results which have shown the effectiveness of psychological interventions in treating substance abuse, the literature has consistently found that in the general population, people often do not seek addiction and mental health services [16]. Potential obstacles to treatment include limited resources, time conflicts, and stigma [17,18,19]. Pregnant individuals, especially individuals belonging to marginalized ethnic and socioeconomic groups, are also more likely to be experience arrest, prosecution, conviction and/or child removal related to substance use disclosure, contributing to increased hesitancy to seek help [20]. Concerns about separation from family, as well as a lack of childcare are also known treatment barriers for pregnant individuals who use substances [21]. 

eHealth is an emerging field that is attracting attention for a variety of mental health conditions. eHealth focuses on the delivery of health services and information through web-based programs, remote monitoring, teleconsultation, and mobile device-supported care. eHealth is a potential avenue to address substance use treatment barriers in pregnancy [22], particularly during COVID-19, which has disrupted a number of face-to-face psychotherapy services. Beyond COVID-19, eHealth initiatives have the potential for broad scale health promotion for substance use [23]. eHealth is accessible, which may make it more appealing to those in remote locations. Additionally, it is cost-effective, and can be flexibly incorporated into one’s schedule [24]. Given the accessible nature of eHealth interventions, some pregnant individuals may prefer to use eHealth interventions as opposed to traditional face to face treatment. Treatment preference is important to consider because matching patients to their treatment preferences has been shown to result in greater reduction of substance use behaviours [25]. Moreover, patient centered care (PCC) is one of the techniques that has been recommended to improve the quality of substance use disorder treatment—and a key aspect of patient centered care is shared decision making [26].

A number of meta-analyses of eHealth interventions for treatment of substance use disorders in the general population have been conducted, with promising results [27,28]; however, the literature in for eHealth interventions treating substance use in pregnancy has yet to be integrated as a review. 

Accordingly, the primary objective of this systematic review and meta-analysis was to evaluate the effectiveness of randomized controlled trials (RCTs) on eHealth interventions delivered during pregnancy with the goal of reducing substance use, where substance use was defined broadly to include any kind of reported alcohol, tobacco, or other drugs. This definition, which includes a variety of substances at varying levels of use was justified by guidelines suggesting that all substance use should be avoided during pregnancy [29]. Substance use was measured by self-reported and objective reports of abstinence. 

## 2. Materials and Methods

Methods outlined by the Cochrane Collaboration’s Handbook [30] and the standards set by Preferred Reporting Items for Systematic review and Meta-Analysis (PRISMA) were used [31,32]. The protocol was registered with Prospero (CRD42020205186) through the University of York Centre for Reviews and Dissemination.

### 2.1. Search Strategy

A preliminary search found that the majority of papers investigating substance use in pregnancy were published in psychology and nursing journals. Thus, we searched the five databases that were most likely to capture the literature within these disciplines. Articles published between 1 January 2000and 19 April 2021were identified from Medline^®^, PsychINFO, EMBASE, CINAHL, and Cochrane CENTRAL. The most recent search took place on 18 April 2021. English-language restrictions were applied. The search terms included database specific controlled vocabulary, field codes, operators, relevant keywords, and subject headings to identify the participant population (pregnant individuals), the exposure (eHealth interventions), and the outcome (substance use) [33,34]. Key terms used to conduct the search were related to telepsychology, randomized control trials, substance use and pregnancy (see Appendix A). Duplicate articles were removed. The remaining articles were divided and were screened independently by two reviewers from a eight member research team. Pairs of reviewers screened the titles and abstracts to determine eligibility for inclusion in the full-text review, and the first author reviewer (KS) supervised and reviewed ~100 records to ensure >85% consistency. Out of 2560 abstracts, 159 conflicts were identified. The types of conflicts included whether the study was targeting the right population (pregnant people), whether the intervention fit our definition for eHealth, or whether the study included extractable outcomes. These conflicts were resolved by the first author (KS).

### 2.2. Inclusion and Exclusion Criteria

Studies were eligible for inclusion if they included: (a) a RCT; (b) an empirical journal article; (c) an eHealth intervention (e.g., video therapy sessions, telephone, SMS, recorded therapy sessions); (d) the goal of the intervention was to reduce substance use; (e) the sample consisted of pregnant individuals; (f) extractable outcomes with respect to substance use; and (g) the intervention took place during pregnancy. If more than one article reported results from the same intervention in the same sample, the more recent study was included in the study. Studies were excluded on the basis that they did not meet inclusion criteria or were irretrievable/unavailable in English.

### 2.3. Data Extraction

Two team members independently extracted data into a Microsoft Excel file and conflicts were resolved by consensus with the coders and the first and second authors. Extracted data included authors’ names, publication years, country, sample demographics, pregnancy characteristics, substance use parameters, intervention characteristics and administration, and mental health assessments for all groups. Sample characteristics that were extracted when provided included sample size, age, gestational age, ethnicity, race, and gender breakdown. Study characteristics that were extracted when provided included the name of intervention, description, method of administration, degree of interactivity (i.e., completely online or some in-person component), degree of guidance, and participant time spent on the intervention. The outcomes extracted were odds ratios (OR) measuring substance use outcomes post-intervention. Corresponding authors of included articles were contacted if studies had missing or incomplete data.

### 2.4. Data Analysis

A random effects meta-analysis was conducted using Comprehensive Meta-Analysis Software (Biostat Inc, Englewood, NJ, USA) [35]. Most studies reported ORs, and these were used to calculate meta-estimates of substance use post-intervention in the intervention groups compared to the control groups. Ref. [36] was the only study to report chi-squares, which were transformed to ORs through the CMA software. Some studies had several post-tests (e.g., immediately post-intervention, later follow-up) and outcomes (e.g., smoking, alcohol use, general substance use). To meet the assumption of independence, effect sizes from the same study were aggregated in CMA and the single effect size estimate for each study was used to calculate pooled ORs. A forest plot was also created to display the ORs for each individual study as well as the pooled OR from all the studies. To test for publication bias, the Begg and Mazumdar rank correlation test [37] as well as the Egger’s regression test [38] were performed to assess bias by regressing standardized effect size to the studies precision. A significant test indicates publication bias, or significant funnel plot asymmetry [36,38,39]. Meta-regression analyses were originally planned to explore significant moderators and explore secondary outcomes; however, not enough studies were included to complete these analyses. Sensitivity analyses were also completed to assess the robustness of the synthesized results.

### 2.5. Quality Assessment

To assess the quality of the RCT studies, the Cochrane Risk of Bias Tool for randomized trials was used [30]. This tool assesses literature based on seven potential sources of bias within the general categories of selection bias (allocation concealment), performance and detection bias (blinding), attrition bias (incomplete data) and selective reporting bias [39]. Bias was judged individually by a team member and then cross-referenced by the judgment of another team member to complete a 100% check. Total scores range from 0 (unlikely to alter the results), to 7 (greatly weakens confidence in the results). Higher scores indicate lower study quality and a higher risk of biased results. The study informally defined scores from 0–2 as low risk, scores from 3–5 as moderate risk, and scores between 6–7 as high risk.

### 2.6. Primary Outcome

The current review aimed to determine whether eHealth interventions delivered during pregnancy reduced substance use when compared to a control group. Substance use was measured using self-reports of frequency and quantity of substances taken, as well as self-report measures of abstinence and objective measures of abstinence. Objective forms of abstinence were defined as a biochemical measure of substance use. For example, in certain studies where smoking was the outcome, carbon monoxide (CO) readings and/or saliva samples were tested for a certain amount of cotinine. 

## 3. Results

### 3.1. Study Selection

**Figure 1:**
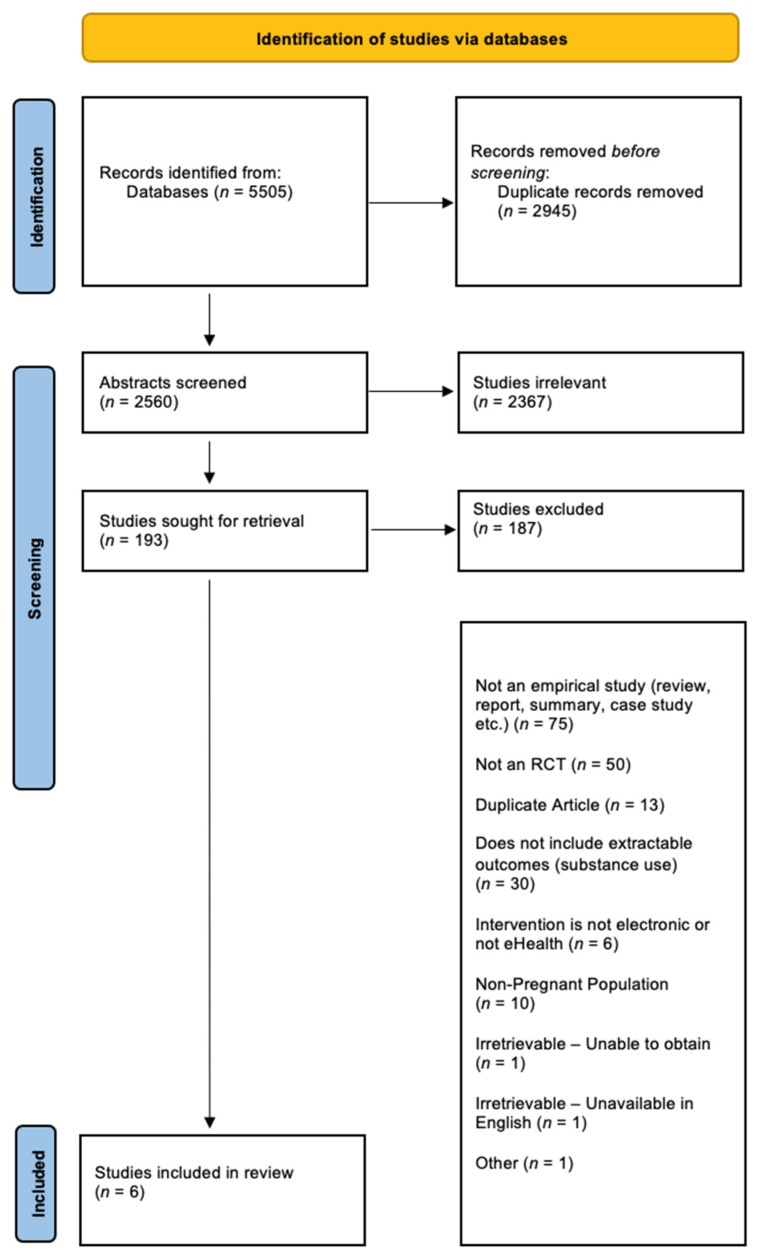
PRISMA diagram detailing the database searches, the number of abstracts screened, and the full texts retrieved.

This search was originally conducted with an associated study [40], which reviewed eHealth interventions in pregnancy for treatment of depression, anxiety, and insomnia. A wider search was conducted to include substance use for the purposes of this paper. The search identified 5505 relevant articles, with 2945 duplicates removed. In total, 2367 of the articles were excluded after title and abstract review and 193 articles were reviewed at the full-text level. A total of 6 articles met inclusion criteria for this review. See Figure 1 for the PRISMA diagram [32]. 

### 3.2. Characteristics of Included Studies

Table 1 provides characteristics of the included studies. Participant baseline age ranged from 18–37 years old. Gestational age ranged from 4–23 weeks. Of the four studies which reported ethnicity, three studies had a total sample where >85% of participants were of European descent [36,41,42].

With respect to the type of eHealth interventions, most of the interventions were created in a way that communication of services took place through the use of technology (i.e., telephone/text), rather than the use of a specific app to reduce substance use behaviours. Four of the eHealth interventions were delivered via computer or the internet [42,43,44,45], one was delivered through text message (SMS) [41] and one was delivered via telephone [36]. The types of interventions that were delivered included: motivational interviewing in one study [43], the use of general health advice (presented educational information regarding substance use without a psychological component) in three studies [42,45,46], and psychoeducation in two studies [36,41]. Three studies assigned control participants to receive treatment as usual from their healthcare providers [41,43,46], one study provided control group participants access to a website with standard advice, [42] and two studies used a time-matched placebo condition [43,44]. 

Interventions varied with respect to whether the eHealth intervention was guided or unguided, which was defined by whether a therapist/healthcare professional facilitated treatment. Most of the included studies were guided (*n* = 4) [36,42,43,44]. Two studies were unguided [41,45]. Length of follow-up varied across studies with follow-up occurring at 4 weeks after baseline [41], 8 weeks after baseline [42], 12 weeks after baseline [43], 16 weeks after baseline [44], up to 22 weeks after baseline [36].

With respect to the type of substances studied, three of the studies assessed smoking using time sensitive self-reported abstinence, as well as dose and dependence tests of drugs [36,41,42]. Two studies assessed alcohol use with time sensitive self-reported abstinence [43,45]. One study measured general substance use using time sensitive self-reported abstinence [44]. Five studies used self-reports of either abstinence or daily substance use behaviours as the outcome [41,42,43,44,45]. Two studies used validated tests of dose and dependence [36,41] carbon monoxide (CO) readings and/or saliva samples were tested for a certain amount of cotinine (<30 mg/mL). One study provided both self-reports of substance use and reports of validated tests of dose and dependence [41]. Only one of the included studies showed a statistically significant benefit of eHealth interventions over the control group [45]. 

### 3.3. Risk of Bias in Included Studies RCTs

**Figure 2:**
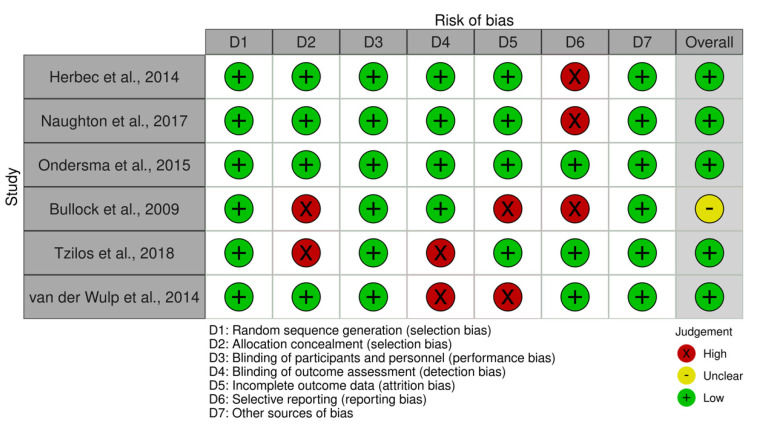
Visual plot demonstrating results from quality assessment. Recall that the study informally defined scores from 0–2 as low risk, scores from 3–5 as moderate risk, and scores between 6–7 as high risk.

The results of the quality assessments are shown in Figure 2. Overall, risk of bias was rated as low for 5 of the 6 studies that were included, where low was defined as a risk of bias score between 0–2. The most common risk of bias was due to attrition (missing data). Other common risks of bias within the current review were detection bias as well as selection bias.

### 3.4. Efficacy of eHealth Interventions on Substance Use

**Figure 3:**
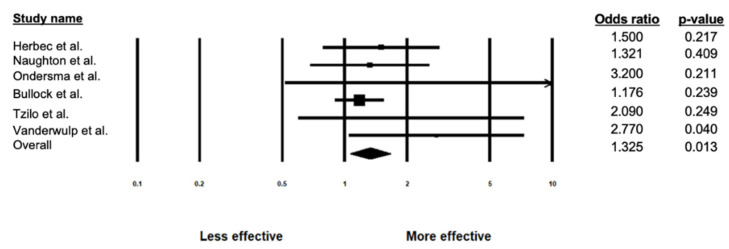
Forest Plot displaying individual and overall effect sizes examining the effectiveness of eHealth interventions.

Using a random-effects model, the efficacy of the eHealth interventions was tested by calculating a pooled OR across 1176 participants and comparing the intervention group (*n* = 585) to control group (*n* = 591). Results showed that pregnant participants who received an eHealth intervention for the treatment of substance use had 1.3 times greater likelihood of reduced substance use compared to those who were assigned to a control group (OR = 1.325, 95% CI = 1.062–1.654, Z = 2.490, *p* = 0.013; see Figure 3 and Table 2). 

**Figure 4:**
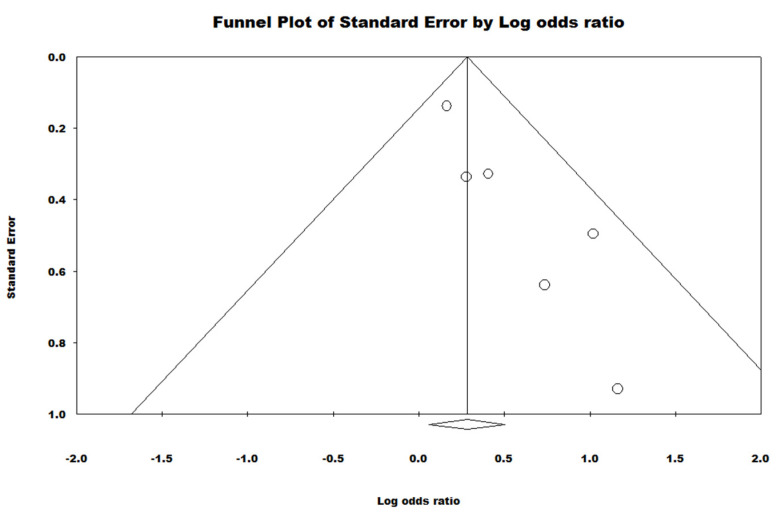
Funnel Plot demonstrating the presence of publication bias.

Significant heterogeneity was observed across studies (Q = 4.505, *p* = 0.479, I^2^ = 0.000). Egger’s regression test (*B* = 1.39, *t*(4) = 4.43, *SE* = 0.324, *p* = 0.012) and the Begg and Mazumdar rank correlation test (*Tau* = 0.600, Z = 1.69, *p* = 0.090) showed mixed findings regarding the presence of publication bias, potentially due to the rank correlation test having lower power [46]. The funnel plot showed clear asymmetry on the positive side which suggests that the overall effect size may be smaller than estimated (See Figure 4). 

A subgroup analysis was also conducted on the studies which measured abstinence (*n* = 4), which also revealed a small size effect size where the odds of increased abstinence was 1.25 times greater for pregnant individuals in the intervention group than for individuals within the control group (OR = 1.251, 95% CI = 0.993–1.577). Although secondary aims of the current study were to evaluate potential moderators of treatment for substance use, not enough studies were identified to conduct a moderator analysis.

### 3.5. Sensitivity Analysis

After systematically removing one study at a time, it was observed that two studies affected the meta-estimate of the effect size of eHealth intervention during pregnancy by more than 5% [36,41]. Specifically, the two studies affected the meta-estimate such that it made the estimate larger [36,41]. When Bullock et al., (2011) was removed, the pooled effect size increased to 1.69 [36]. When Naughton et al., was removed, the pooled effect size increased to 1.41 [41]. 

## 4. Discussion

### 4.1. Primary Findings

The current systematic review and meta-analysis synthesized existing evidence from 6 RCTs on the efficacy of eHealth interventions for substance use among a pregnant population by comparing participants using an eHealth intervention (*N* = 585) to participants in a control group (*N* = 591). Participant ages ranged from 18–37 years old and gestational ages ranged from 7.73–14.7 weeks. All the studies took place in an economically advantaged country which speaks to the potential difference in the accessibility for eHealth interventions for developing countries [47]. Most of the included RCTs measured smoking and drinking outcomes, whereas studies on eHealth interventions for harder drug use were more uncommon. The lack of studies measuring harder drug use among this population may be due to the stigma associated with these substances—particularly during pregnancy [48]. The majority of the eHealth interventions were delivered via computer/internet which is consistent with other reviews on eHealth where most eHealth interventions were internet-based. This may be attributed to the rise in technology use in recent years and that most of the included studies recently took place between the years of 2014–2018. With respect to the types of interventions used, most of the included interventions were brief in nature and had minimal clinician guidance, which could have reduced the current effect sizes. There was also variability in the time of follow-up among studies, with some studies having a shorter follow-up ranging from 4 weeks to 6 months. All of the included studies were assessed to have a low risk of bias which provides support for the high quality of the studies in the current review. The most common type of bias noted among studies was attrition bias, though this is common for eHealth interventions [49].

Results of the meta-analysis showed that eHealth interventions delivered in pregnancy reduced substance use when compared to control conditions (OR = 1.33, *p* = 0.013). This effect size was calculated using a predominantly intent-to-treat sample (ITT). With the exception of one study [43], each of the included studies reported an OR which were included based on an ITT analysis. Though it should be noted that the study which did not use an ITT sample completed a sensitivity analysis and found that the completers sample as opposed to the use of an ITT sample did not significantly differ [43]. Results suggest that eHealth interventions are significantly more effective than control conditions in reducing substance use in pregnancy. Findings from the sensitivity analyses found that two studies when removed, made the effect size larger [36,41]. Of note, both of these studies had smoking as an outcome which suggests that smoking may be less amenable to treatment in comparison to other substances. Furthermore, both the [36] study and the [41] study used a telephone to deliver their intervention. This is in comparison to the other studies which largely relied on the computer/internet. This may suggest that telephone and or text interventions may not be as effective in comparison to other modes of eHealth delivery.

### 4.2. Consistency with Existing Literature

The finding of a small yet significant effect of eHealth interventions is consistent with current literature of eHealth interventions for substance use within non-pregnant populations [27,50]. For example, in a meta-analysis examining the effectiveness of internet interventions for adults with substance abuse issues, results showed that internet interventions significantly decreased substance use with a small to moderate effect size (*Hedge’s g* = 0.36) [27]. Results are also consistent with a meta-analysis examining the effectiveness of internet interventions for adult alcohol misuse, which also found a small but significant effect size in favour of the internet interventions (*Hedge’s g* = 0.20) [50]. With respect to the effectiveness of eHealth interventions in a pregnant population, no review to date has synthesized this information. However, one review has observed the effectiveness of technology-based interventions for substance use among participants who were of a child-bearing age (ages 18–45) [51]. Results from this meta-analysis found that technology-based interventions were efficacious in comparison to control groups in preventing and reducing substance use for individuals at a child-bearing age, though the effect size was small (*d* = 0.19) [51] which is in line with the current review.

The effect sizes of eHealth interventions are generally consistent with those of face-to-face interventions for substance use within the general population. For example, a meta-analysis of psychosocial interventions for substance use in the general population found a significant yet small to moderate effect size for treatment (*Cohen’s d* = 0.45) [28]. In another meta-analysis evaluating the effectiveness of motivational interviewing on substance use behaviours in adolescence, interventions were effective in improving substance use outcomes, but the effect sizes were again small (*Cohen’s d* = 0.17) [52].

### 4.3. Obstacles to eHealth Interventions

In the studies included in this review, there were high attrition rates and varied engagement levels [53,54]. The observed attrition rates varied widely from 2% [44] to 33.5% [42] which is consistent with other eHealth interventions which typically range from 19% to 50% [55,56]. Individuals with substance use problems are more likely to terminate treatment than individuals with other psychosocial problems, with substance use treatment programs reporting the highest dropout rates when compared to individuals with other psychosocial concerns [57]. Taken together, these findings suggest that individuals taking part in a substance use eHealth intervention may require additional guidance and human monitoring to decrease levels of dropout. In the current review, only one study involved human monitoring, though this did not appear to increase effect sizes over and above other studies [36].

Furthermore, there were relatively high levels of participant attrition within the reviewed studies. This is a common feature among substance use eHealth studies and may in part be due to the lack of human monitoring. Across all eHealth programs, retaining pregnant participants to enhance positive outcomes continues to be a challenge. This speaks to the need to evaluate different ways to reduce attrition by improving participant engagement in treatment (e.g., gamification) [49,58] and implementing more rigorous designs which could include human monitoring and follow-ups by clinicians to reduce the high participant dropout rates. For example, the one study which did implement human monitoring had lower attrition rates relative to most of the other included studies [36]. In doing this, future eHealth interventions for substance use would maximize its clinical effectiveness [54,59].

### 4.4. Strengths, Limitations and Future Directions

Prior systematic reviews and meta-analyses were limited by the population studied, which to date, have only included the effects of eHealth on substance use within a general population. While some reviews exist on the effect of eHealth interventions on participants of “child-bearing age,” these participants are not recruited during pregnancy and do not complete the eHealth intervention during pregnancy. The current study is the first, to our knowledge, to have synthesized the findings from the literature within a specifically pregnant population. This is incredibly important because as mentioned previously, many mental health and substance use behaviours in pregnancy persist into the postpartum period. Moreover, substance use has unique negative effects during pregnancy and pregnant populations may require support that is tailored to their needs.

Despite these strengths, the findings from this meta-analysis should be interpreted in the context of several limitations. Notably, the current review included a small number of studies (*n* = 6). Due to the limited number of studies included in the current analysis, there was not enough statistical power to conduct moderator analyses. Future research should investigate if demographic variables and/or study characteristics moderate the relationship between eHealth interventions and substance use during pregnancy. A larger research base is needed to better understand specifically what types of eHealth interventions and methods of delivery are most effective in pregnancy, and for whom. Substance use outcomes also varied—where most of the included studies assessed smoking cessation. Consequently, future reviews may benefit from sub-group analyses to investigate if the effectiveness of eHealth interventions vary as a function of the type of substance use being treated and/or differences in using self-report and objective measures to assess treatment success. There was also wide variability in the types of eHealth interventions that were used, with delivery occurring through internet, phone, and text messages, which likely have also influenced the heterogeneity of the reported outcomes in this review. It should also be noted that since all reviewed studies took place in economically advantaged countries (i.e., United States, England, Netherlands), the lack of studies from other countries may limit the generalizability of our findings. Additionally, publication bias was observed in the outcomes Egger’s Regression test (*p* = 0.012), which may suggest that effect sizes were over-estimated within the current review [38].

Future interventions should consider the high comorbidity that substance use has with various mental health concerns, including anxiety [60], depression [60], bipolar disorder, [61], attention-deficit hyperactivity disorder [62], and suicidality [63,64]. Moreover, individuals in the general population who were treated in programs providing specific treatment to target concurrent disorders had higher rates of using mental health services, which predicted improvements in both mental health and substance use following treatment [65]. Lastly, future studies should also examine the impact of eHealth treatments during pregnancy and in the postpartum period and beyond, with the hope that eHealth interventions will be able to create long-lasting treatment effects which persist beyond the intervention period.

### 4.5. Clinical Implications

According to the World Health Organization, all pregnant people should have access to affordable treatment options that respects their autonomy [66]. eHealth interventions may be best used as a first line intervention in stepped care models, as they may increase accessibility for some clients, and be less costly than more intensive in-person approaches [67,68]. The privacy and anonymity afforded by eHealth interventions may increase the likelihood of seeking support for substance use in pregnancy. eHealth interventions are also in line with international guidelines for the treatment of substance use during pregnancy [66,69]. eHealth interventions have the potential to be tailored to track substance use and treatment progress, as well as provide information on where pregnant people can receive more intensive substance treatment, which is in line with the National Institute for Health and Care Excellence guidelines in the treatment of substance use during pregnancy [69].

However, it is important to recognize that not everyone has access to reliable internet. Indeed, according to a report from the United Nations, over half of the world population does not have access to reliable internet, and there are important sociodemographic disparities in internet access within countries [70]. For example, only 24% of First Nation reserves in Canada have access to reliable internet [71]. Moreover, internet use may be limited be lack of devices (i.e., only one computer for one family), and compromised internet speed due to multiple devices. In another study which looked at telemedicine use in Peru, noted that almost 60% of the population in Peru belong to the lowest socioeconomic strata, preventing them from owning devices such as a computer or smartphone with internet access [72]. Furthermore, due to the lack of owning these devices, these individuals may lack the technological skills to know how to access and utilize telemedicine services, even if a device is provided to them [72]. Additionally, in a study of the disparities in digital access among Medicare beneficiaries, results found that individuals who lacked digital access were higher among those with low socioeconomic status, those 85 years or older, and in certain ethnic communities [73].

Accordingly, improved internet coverage and digital access has been highlighted as an important step into making eHealth more accessible. The future of eHealth should include determining how these interventions can be properly incorporated into the current healthcare system to increase patient accessibility to mental health services.

## 5. Conclusions

Taken together, the review found that eHealth interventions are effective in treating substance use during pregnancy. Furthermore, eHealth interventions are a promising healthcare intervention that increase access to care. In order to increase effect sizes, adaptations should be considered with development occurring, in conjunction with patient and provider partners. For example, future eHealth interventions could be more integrated such that treatment is completed in conjunction with therapist or peer support and additional guidance could be provided by having increased interactions with clinicians as opposed to pre-set modules which are to be completed at the patient’s pace. This suggestion is supported by the finding that guided eHealth interventions were significantly superior to unguided interventions [74].

## Figures and Tables

**Table 1:** Study Characteristics.

References	Title	Year	Country	Intervention (N); Control (N)	Intervention, Age of Sample (M, SD), Range (y)	Control, Age of Sample (M, SD), Range (y)	Intervention, Gestational Age (M, SD), Range (w) Control, Gestational Age (M, SD), Range (w)	Type of eHealth Intervention	Description of Intervention	Time of Follow Up
Bullock, L., et al. [36]	Baby BEEP: A Randomized Controlled Trial of Nurses’ Individualized Social Support for Poor Rural Pregnant Smokers	2009	United States	Telephone Social Support Group and Booklet Group (117), Control (119)	Telephone Social Support Group and Booklet Group (23.1, 4.3) Telephone Social Support Only Group (24.0, 4.7) Booklet Only Group (23.6, 4.8)	Control (23.9, 4.8)	13.5 weeks 13.5 weeks	Telephone Intervention	Baby BEEP intervention consisted of a scheduled weekly telephone call and 24-h access to the nurse for any additional social support needed	T2 (28–32 weeks gestation; T3: 6 weeks postpartum)
Naughton, F. [41]	Large multi-centre pilot randomized controlled trialtesting a low-cost, tailored, self-help smoking cessationtext message intervention for pregnant smokers (MiQuit)	2017	England	203 204	26.6 (5.7), 16.9–40.0	26.4 (5.7), 16.6–41.3	14.6 weeks (4.2), 4–23 14.7 weeks (4.5), 3–24	Text Message Based Intervention	MiQuit is an automated text support service that delivers information or motivational messages	4 weeks post-randomization up until 36 weeks gestation
Herbec, A., et al. [42]	Pilot randomized controlled trial of an internet-based smoking cessation intervention for pregnant smokers (‘MumsQuit’)	2014	England	99 101	27.6 (6.0)	26.1 (5.8)	NR	Internet-Based Intervention	MumsQuit is a personalized, interactive quitting plan that mimics advisory support from a smoking cessation expert	8 weeks post baseline
Ondersma, S. J., et al. [43]	Computer-Delivered Screening and Brief Intervention for Alcohol Use in Pregnancy: A Pilot Randomized Trail	2015	United States	24 24	18–25 (50.0%) 26–33 (33.3%) 34–37 (16.7%)	18–25 (58.3%) 26–33 (33.3%) 34–37 (8.3%)	12.5 weeks (5.6) 12.0 weeks (5.3)	Computer-delivered screening and brief intervention (eSBI)	A brief 20-min video was delivered via tablet while waiting for a prenatal care appointment and three separated tailored mailings followed	3 month follow up
Tzilos, Wernette. G., et al. [44]	A Pilot Randomized Controlled Trial of a Computer-Delivered Brief Intervention for Substance Use and Risky Sex During Pregnancy	2018	United States	31 19	25.1 (5.79)	23.2 (4.21)	12.9 (4.76) 13.9 (4.21)	Computer Delivered Intervention	A single motivational session and a booster session	4 month follow up
van de Wulp, N., et al. [45]	Reducing Alcohol Use During Pregnancy Via Health Counseling by Midwives and Internet-Based Computer-Tailored Feedback: A Cluster Randomized Trial	2014	Netherlands	Computer tailoring (111); Usual care (124)	32.31 (4.22)	33.53; (3.85)	7.73 weeks (2.06) 7.92 weeks (1.99)	Computer delivered intervention	Respondents in the computer-tailoring group received usual care from their midwife and computer-tailored feedback via the Internet. This feedback was tailored to the participant’s alcohol use, knowledge, risk perception, attitude, social influence, self-efficacy, intention, and action and coping plans	6 months after baseline

**Table 2:** Primary Outcomes.

References	Title	Year	Type of Substance	Measurement used for Assessment	Intervention Effect on Substance Use	Odds Ratio	Confidence Interval	*p*-Value	Quality Assessment Rating
Bullock, L., et al. [36]	Baby BEEP: A Randomized Controlled Trial of Nurses’ Individualized Social Support for Poor Rural Pregnant Smokers	2009	Smoking	Readiness to Stop Smoking, The Fagerstrom Test for Nicotine Dependence and Dosage (cotinine < 30ng/mL)	The nurse-delivered social support telephone intervention was not more effective than booklets alone or usual care in reducing smoking behaviour.	1.18	0.90–1.54	0.239	3
Naughton, F. [41]	Large multi-centre pilot randomized controlled trial testing a low-cost, tailored, self-help smoking cessation text message intervention for pregnant smokers (MiQuit)	2017	Smoking	Validated 4-week continuous abstinence (CO readings < 9ppm), Self-reported 4-week continuous abstinence, 7-day point prevalence for 4-weeks continuous abstinence	No statistical significance was found between the MiQuit intervention group and the usual care control group.	1.32	0.68–2.56	0.409	1
Herbec, A., et al. [42]	Pilot randomized controlled trial of an internet-based smoking cessation intervention for pregnant smokers (‘MumsQuit’)	2014	Smoking	Self-reported 4-week continuous abstinence	The analysis determined no significant difference when measuring continuous abstinence rates between the MumsQuit intervention and control group.	1.50	0.79–2.86	0.217	1
Ondersma, S. J., et al. [43]	Computer-Delivered Screening and Brief Intervention for Alcohol Use in Pregnancy: A Pilot Randomized Trail	2015	Alcohol Use	Self-reported 90-day abstinence period	No statistical significance was found between the intervention group and the control group when comparing abstinence.	3.20	0.52–19.78	0.211	0
Tzilos, Wernette. G., et al. [44]	A Pilot Randomized Controlled Trial of a Computer-Delivered Brief Intervention for Substance Use and Risky Sex During Pregnancy	2018	General substance use	Self-reported substance use behaviours using a calendar and multiple prompts	The final analysis determined no significant reduction of substance use in the intervention group compared to the control group.	2.06	0.59–7.31	0.249	2
van de Wulp, N., et al. [45]	Reducing Alcohol Use During Pregnancy Via Health Counseling by Midwives and Internet-Based Computer-Tailored Feedback: A Cluster Randomized Trial	2014	Alcohol	Average alcohol consumption during pregnancy was assessed with the 5-item Dutch Quantity-Frequency-Variability (QFV) Questionnaire	The final analysis showed that computer-tailoring respondents used alcohol significantly less often when compared to usual care respondents.	2.77	1.05–7.32	0.040	2

## Data Availability

Not applicable.

## Appendix A

**Table A1:** Terms used in Search Strategy.

eHealth/Telepsychology	And	Study Design	And	Mental Health and Substance Use	And	Pregnancy
eHealth/e-Health		RCT		Substance Use		pregnant
internet		efficacy		externalizing		perinatal/peri-natal
online/on-line		random allocation		substance-related disorder/substance related disorder		prepartum/pre-partum
app/apps		effectiveness		substance abuse/ substance-abuse		antenatal/ante-natal
web-based/web based		randomized controlled trial		substance dependence/substance-dependence		birth
smart-phone/smartphone/smart phone		trial		addiction		childbirth/child-birth
mobile phone/mobile-phone		controlled clinical trial		drug abuse/drug-abuse		labor
mobile health		clinical trial		drug dependence/ drug-dependence		labour
mHealth				alcohol abuse/alcohol-abuse		gestation
app-based				alcohol dependence/alcohol-dependence		
computer systems				alcoholism/alcoholic		
computers						
cell phone/cell-phone/cellphone						
website						
computer						
social media						
web-based/web based						
SMS						
mobile						
text-based/text based						
digital						
self-directed/self directed						
technology-assisted/technology assisted						
self-help/self help						
self-guided/self guided						
telecommunications/telecommunication						
